# Insights Into the Emerging Role of Baf53b in Autism Spectrum Disorder

**DOI:** 10.3389/fnmol.2022.805158

**Published:** 2022-02-03

**Authors:** Megan E. Rowland, Jana M. Jajarmi, Tess S. M. Osborne, Annie Vogel Ciernia

**Affiliations:** Department of Biochemistry and Molecular Biology, Djavad Mowafaghian Centre for Brain Health, The University of British Columbia, Vancouver, BC, Canada

**Keywords:** gene expression, chromatin remodeling, BAF complex, long-term memory, synaptic plasticity

## Abstract

Accurate and precise regulation of gene expression is necessary to ensure proper brain development and plasticity across the lifespan. As an ATP-dependent chromatin-remodeling complex, the BAF (Brg1 Associated Factor) complex can alter histone-DNA interactions, facilitating dynamic changes in gene expression by controlling DNA accessibility to the transcriptional machinery. Mutations in 12 of the potential 29 subunit genes that compose the BAF nucleosome remodeling complex have been identified in several developmental disorders including Autism spectrum disorders (ASD) and intellectual disability. A novel, neuronal version of BAF (nBAF) has emerged as promising candidate in the development of ASD as its expression is tied to neuron differentiation and it’s hypothesized to coordinate expression of synaptic genes across brain development. Recently, mutations in BAF53B, one of the neuron specific subunits of the nBAF complex, have been identified in patients with ASD and Developmental and epileptic encephalopathy-76 (DEE76), indicating BAF53B is essential for proper brain development. Recent work in cultured neurons derived from patients with BAF53B mutations suggests links between loss of nBAF function and neuronal dendritic spine formation. Deletion of one or both copies of mouse Baf53b disrupts dendritic spine development, alters actin dynamics and results in fewer synapses *in vitro.* In the mouse, heterozygous loss of Baf53b severely impacts synaptic plasticity and long-term memory that is reversible with reintroduction of Baf53b or manipulations of the synaptic plasticity machinery. Furthermore, surviving Baf53b-null mice display ASD-related behaviors, including social impairments and repetitive behaviors. This review summarizes the emerging evidence linking deleterious variants of BAF53B identified in human neurodevelopmental disorders to abnormal transcriptional regulation that produces aberrant synapse development and behavior.

## Introduction

Autism Spectrum Disorder (ASD) is a common neurodevelopmental disorder characterized by deficits in communication and social interactions, as well as by repetitive or restricted behaviors and intellectual disability (ID) (Sharma et al., 2018; Lord et al., 2020). In addition to these core symptoms, there are several co-occurring neuropsychiatric disorders or symptoms including hyperactivity, attention disorders, anxiety, depression, and epilepsy (Lord et al., 2020). The rising diagnosis rates of ASD and limited treatments currently available, make the need to accelerate the discovery and development of novel therapeutics of critical importance. While ASD is highly heritable, the genetic architecture is complex, with hundreds of genes implicated and any single gene mutation accounting for only 0.5–2% of ASD cases (Devlin and Scherer, 2012). The majority of ASD risk genes are critical for synapse formation and function, or chromatin remodeling and gene regulation (Lord et al., 2020).

Within eukaryotic cells, nuclear DNA is packaged in chromatin. The basic unit of chromatin is the nucleosome, in which DNA is wrapped around an octamer of histone proteins. Access to the underlying DNA is controlled by regulation of nucleosome-DNA interactions. Chromatin remodeling complexes use the energy of ATP to slide, evict, or restructure nucleosomes in concert with other regulatory factors to control access to the DNA and ultimately gene expression (Clapier and Cairns, 2009). There are four families of ATP-dependent chromatin remodelers—ISWI, SWI/SNF (BAF), CHD, and INO80 (Clapier et al., 2017). Mammalian BAF (BRG1/BRM associated factor) complexes are multisubunit ATP dependent chromatin remodelers that play essential roles in human development and disease (Lessard et al., 2007; Zahir and Brown, 2011; Staahl and Crabtree, 2013; Kadoch and Crabtree, 2015; Ciernia et al., 2017; Sokpor et al., 2018; Alfert et al., 2019; Cyrta et al., 2020). Mutations in the BAF complex are associated with neurodevelopmental disorders including ASDs (Devlin et al., 2012; O’Roak et al., 2012; De Rubeis et al., 2014; Vogel-Ciernia and Wood, 2014; Wenderski et al., 2020), schizophrenia (Koga et al., 2009; Vogel-Ciernia and Wood, 2014), Coffin-Siris syndrome (Santen et al., 2013; Wieczorek et al., 2013), Nicolaides-Baraitser syndrome (Van Houdt et al., 2012; Wieczorek et al., 2013), and Kleefstra’s syndrome (Kleefstra et al., 2006). The BAF complex is critical for proper brain development (Lessard et al., 2007; Zhan et al., 2011; Vogel-Ciernia et al., 2013; Petrik et al., 2015), and BAF complex subunit mutations are predicted to have deleterious impacts on neuronal development and function.

Neuronal activity induces the expression of genes that are required for synaptic function and plasticity. Neuronal depolarization produces a rapid increase in intracellular calcium which initiates a cascade of downstream signaling molecules resulting in the activation of gene expression. For instance, during a novel learning event, neuronal firing induces expression of immediate early genes (IEGs). IEGs are rapidly upregulated upon neuronal activity and often used as a surrogate to identify active neuronal ensembles (Guzowski et al., 1999; Gallo et al., 2018). A prime example of this is *c-fos*, an IEG whose rapid expression following behavioral training correlates with learning and cellular programs involved in memory retrieval (Guzowski et al., 2001; Gallo et al., 2018). Many IEGs are transcriptional regulators themselves and their expression initiates a cascade of cellular remodeling events that culminates in the strengthening and weakening of specific synaptic connections within brain circuits thought to underlie learned behaviors. The formation and plasticity of synapses are mediated by alterations in cytoskeletal actin dynamics (Rex et al., 2009; Chen et al., 2007, 2010), where actin polymerization facilitates the outgrowth of synapses. Mutations in synaptic genes are also commonly observed in ASD and other neurodevelopmental disorders. Together, this suggests that genetic mutations that disrupt transcription regulation or cause synapse dysfunction may converge to produce overlapping negative impacts on brain development.

The BAF complex appears to be critical for neuronal dendrite growth, synapse development, and several behaviors relevant to both ID and ASD. Despite the evidence that mutations in the BAF complex are connected to ASD (Lopez and Wood, 2015), it is still unclear how mutations in the BAF complex actually lead to ASD. Within the last 4 years, several reports have been published describing mutations in the BAF complex subunit Baf53b in the neurodevelopmental disorders Developmental and epileptic encephalopathy-76 (DEE76) and ASD (Bell et al., 2019; Fichera et al., 2019; Maddirevula et al., 2019; Yüksel et al., 2019; Wenderski et al., 2020). This review will summarize the data from these recent reports while shedding light on the role of the BAF complex in neuronal gene expression and how this might contribute to symptoms of patients with Baf53b-induced neurodevelopmental disorders.

## BRG1/BRM Associated Factor Complex

The BAF (mSWI/SNF) complex is a 1–1.5 MDa complex with a highly diverse subunit composition (i.e., many paralogs within the complex), which provides cell type and tissue specificity in mammals (Mashtalir et al., 2018). The SWI/SNF complex was first discovered in yeast, and homologous chromatin remodeling complexes were also found in drosophila (Brahma-associated protein (BAP) complex), and eventually in mammals (BAF). The complexity of the BAF complex relative to BAP and SWI/SNF homologs is evident when comparing subunit conservation between organisms. Critical functional subunits are conserved between mammals and yeast, such as BAF250a/b (Swi1), Baf47 (Snf5), Baf155 (Swi3), BAF60 (Swp73), BAF53 (ARP7/9), and Baf45 (Swp 82) (Alfert et al., 2019; Marcum et al., 2020). Newly evolved subunits BCL7a/b/c, BCL11a/b, BRD7/9, and SS18/CREST do not have homologs in yeast (Alfert et al., 2019). The complexity of the BAF complex increases with the genomic complexity of the organism (Wang et al., 1996). In yeast, the SWI/SNF complex mostly activates transcription and in Drosophila, the main function of BAP is to oppose the polycomb repressive complex (PRC) (Alfert et al., 2019). Conversely, the mammalian BAF complex is able to both activate and repress genes and its function can vary depending on the need of the cell (Alfert et al., 2019).

The mammalian BAF complex is made up of 15 subunits which assemble in a combinatorial fashion, with specific combinations depending on both the cell type and developmental stage (Wu et al., 2007; Staahl and Crabtree, 2013; Kadoch and Crabtree, 2015). The ATPase subunit is either BRG1 or BRM (Son and Crabtree, 2014), but across different cell types, remaining subunit composition of BAF changes. The exchange of cell type specific subunits during development confers unique targets and functions of BAF. The polymorphic nature of the BAF complexes give rise to hundreds of different complexes variations. For example, the embryonic stem cell BAF (esBAF) complex controls gene expression important for pluripotency. Similarly, neural progenitor BAF (npBAF) maintains neural stem cell identity, and the neuronal BAF (nBAF) contains subunits essential for neuronal development and function (Yoo et al., 2009; Bao et al., 2013; Staahl and Crabtree, 2013; Vogel-Ciernia et al., 2013; Vogel-Ciernia and Wood, 2014; Kadoch and Crabtree, 2015; Lopez and Wood, 2015; López et al., 2020).

Cell type specificity of BAF is driven by correspondingly specific expression of distinct subunits. The neuron specific subunits of nBAF are expressed exclusively in neurons (Harata et al., 1999; Olave et al., 2002). In neuronal progenitors npBAF has the unique subunits Baf53a, Baf45a, and SS18. During neuronal differentiation these subunits are exchanged for Baf53b (also known as Actl6b), Baf45b/c, and CREST (Staahl et al., 2013). This subunit switching drives cell cycle exit of neural progenitors, allowing for neuron specific gene expression and functions such as axonal guidance and dendritic branching (Aizawa et al., 2004; Wu et al., 2007; Staahl and Crabtree, 2013; Braun et al., 2021). The nBAF specific subunits appear to confer unique functions to nBAF. For example, CREST is a calcium binding protein that upon neuronal stimulation helps target nBAF to neuron specific genes required for dendritic outgrowth (Aizawa et al., 2004). The function of Baf45b remains elusive, although it has been suggested that Baf45b requires other neuronal subunits and/or specific neuronal post-translational modifications for its full function (Lessard et al., 2007). The majority of this review will focus on Baf53b, the most highly studied neuron-specific nBAF subunit that has been critically linked to neuronal development, plasticity and ASD (Vogel-Ciernia and Wood, 2014).

## Non-Redundant Functions of BAF53A and BAF53B

Baf53b is a class 4 actin-related protein (Arp) that is expressed exclusively in neurons (Harata et al., 1999; Park et al., 2002). Arps are structurally similar to actin at their ATP binding pockets and actin folds, however, divergent surface features of Arps may produce distinct functions. A novel human Arp, Baf53a, was found to be a subunit of the BAF complex that disrupts nucleosomes *in vitro* (Zhao et al., 1998). Harata et al. (1999) identified Baf53b based on its sequence similarity to Baf53a. Baf53b and Baf53a are 83% identical based on their amino acid sequence, with the most divergent region appearing in subdomain 2 (SB2) from amino acids 39 to 82 (Harata et al., 1999; Wu et al., 2007). Both Baf53a and Baf53b contain nuclear localization signals and they localize entirely to the nucleus in regions of euchromatin (Harata et al., 1999). Analysis of total RNA fractions obtained from samples from a wide variety of human tissues showed that Baf53b was expressed exclusively in the brain and neuronal tissues, while Baf53a was expressed in all tissues except the brain (Harata et al., 1999; Kuroda et al., 2002; Park et al., 2002). Baf53b is combinatorially assembled into nBAF complexes and interacts strongly with both BRM and BRG1 ATPase subunits (Olave et al., 2002), but is not required for nBAF complex assembly as all 10 core subunits assemble in the absence of Baf53b (Wu et al., 2007; Wenderski et al., 2020). Additionally, Baf53b does not have any known nBAF complex-independent functions and Baf53b is not found in complexes other than nBAF (Lessard et al., 2007; Wu et al., 2007; Staahl et al., 2013).

Baf53a and Baf53b appear to have largely non-overlapping and non-redundant roles in BAF complexes. The switch from Baf53a to Baf53b upon neuronal differentiation is highly controlled through selective expression of miR-9* and miR-124, which silence expression of Baf53a, mediating cell cycle exit of neural progenitor cells (Yoo et al., 2009). Baf53a appears uniquely critical for npBAF’s function. Neural progenitors do not proliferate properly if Baf53b is exchanged for Baf53a in npBAF (Wu et al., 2007). However, Baf53b does promote the survival of neuroprogenitors in the absence of Baf53a (Zhu et al., 2017). In the context of nBAF, there is conflicting data as to the level of compensation Baf53a can confer in the absence of Baf53b. In the initial characterization of Baf53b knockout neurons, Baf53a was not found to be retained in nBAF complexes lacking Baf53b (Wu et al., 2007). However, in subsequent follow up work, Baf53a was found to be fully retained in Baf53b^–/–^ nBAF complexes, however, these complexes were not as stably associated with neuronal chromatin (Wenderski et al., 2020). In either case, Baf53a is unable to functionally compensate for a lack of Baf53b in the context of neuronal development (Wu et al., 2007). Despite being 83% identical, exchanging Baf53a for Baf53b in the nBAF complex failed to rescue neuronal dendritic branching deficits observed in Baf53b^–/–^ cultures (Wu et al., 2007). Comparable experiments with Baf45a and Baf45b showed similar results (Lessard et al., 2007).

## Patient Mutations in *BAF53B*

**FIGURE 1:**
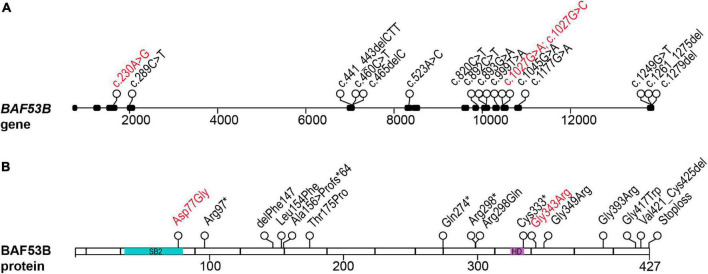
Patient mutations in the BAF53B gene. **(A)** The BAF53B gene locus. Each circle represents a nucleotide position where a mutation was found by whole exome sequencing. Black boxes represent exons and nucleotide position of the mutation is in the coding sequence (c.). Scale is in base pairs. **(B)** The BAF53B protein. Each circle represents an amino acid position where a mutation was found by whole exome sequencing. The most divergent regions of BAF53B, SB2, is located in amino acids 39–82 (blue). The HD, predicted to form protein-protein interactions with other nBAF subunits, is located in amino acids 323–333 (purple). Scale is in amino acids. Recessive mutations are in black text and dominant mutations in red text.

Recently, mutations in *BAF53B* have been identified in patients exhibiting Developmental and epileptic encephalopathy-76 (DEE76; OMIM #618468). DEE76 is an autosomal recessive neurodevelopmental disorder characterized by seizures, developmental delay, intellectual disability and delayed myelination. Karaca et al. (2015) first identified two siblings of consanguineous parents presenting with severe intellectual disability, microcephaly, seizures, and autistic behaviors. The origin of their unknown developmental disorder was attributed to a mutation in exon 10 of *BAF53B* (c.893G > A; p.Arg298Gln) (Karaca et al., 2015). Several years later, four papers were published further implicating mutations in *BAF53B* in neurodevelopment disorders and until this time *BAF53B* had not been conclusively reported to play a role in disease (Bell et al., 2019; Fichera et al., 2019; Maddirevula et al., 2019; Yüksel et al., 2019; Figure 1). In the most comprehensive study, 8 families were identified by whole exome sequencing; children with *BAF53B* mutations had global developmental delay, seizures, intellectual disability, and delayed myelination (Bell et al., 2019). Several homozygous recessive and compound heterozygous mutations were found in *BAF53B* (Bell et al., 2019; Figure 1 and Table 1). Of the recessive mutations, none were found in the SB2 domain and they did not cluster in any particular area of *BAF53B* (Bell et al., 2019). Of note, a mutation in exon 12 (c.1045G > A;p.Gly349Ser) was found in a highly conserved glycine residue in two unrelated children by Bell et al. (2019) and one child by Fichera et al. (2019). It is predicted that most of the recessive mutations identified are non-sense or frameshift mutations and result in non-sense mediated decay of the transcript, as they occur before the final exon of *BAF53B*. Heterozygous *de novo*, dominant missense mutations were discovered in 10 patients who displayed hypotonia, intellectual disability, developmental delay, autism, and Rett-like stereotypies (Bell et al., 2019). Of the dominant mutations, 8 children from different families had the mutation c.1027G > A (p.Gly343Arg) present in exon 12 (Bell et al., 2019). Additionally, one child was identified with a mutation in the SB2 domain (c.230A > G;p.Asp77Gly) (Bell et al., 2019; Figure 1 and Table 1).

**TABLE 1:** Summary of Baf53b mutations and patient symptoms.

Coding change	Protein change	Mutation type	Citation	Age of assessment	Sex	ASD	Intellectual disability	Epilepsy	Develop- mental delay	Hypotonia	Myelin abnormalities	Lethal	Un- diagnosed, potentially affected siblings	Protein information
c.230A > G	p.Asp77 Gly	Dominant, *de novo* missense	Bell et al., 2019	8 years	Female	Yes	Yes	Yes	Yes	No	No	Unknown	No	Subdomain 2
c.289C > T	p.Arg97*	Homozygous recessive	Bell et al., 2019	4 years	Female	No	Yes	Yes	Yes	Yes	No	Unknown	No	
c.389G > A, c.556C > T	p.Arg130Gln, p.Gln186*	Recessive, compound heterozygous	Bell et al., 2019	8 years	Female	No	Yes	Yes	Yes	Yes	Yes	Unknown	No	
c.441_443 delCTT	p.del Phe147	Homozygous	Bell et al., 2019	3 years	Female	No	Yes	Yes	Yes	Yes	Yes	Unknown	No	
c.460C > T	p.Leu154 Phe	Homozygous missense	Wenderski et al., 2020	3 years (Female)	2 males, female	Yes	No	Yes	No	No	Yes	Unknown	No	
c.465delC	p.Ala156 Profs*64	Homozygous truncating	Wenderski et al., 2020	4 months	Male	Yes	No	Yes	No	No	Yes	Unknown	No	
c.523A > C	p.Thr175 Pro	Homozygous missense	Wenderski et al., 2020	3 years	Male, female	Yes	Yes	Yes	Yes	No	Yes	Unknown	No	
c.617T > C, c.724C > T	p.Leu206Pro, p.Gln242*	Recessive, compound heterozygous	Bell et al., 2019	14 months	Female	No	Yes	Yes	Yes	Yes	Yes	Unknown	No	
c.695delC, c.1275C > A	p.Pro232 Glnfs*24, p.Cys425*	Recessive, compound heterozygous	Bell et al., 2019	5 years	Male	No	Yes	Yes	Yes	Yes	No	Yes	No	
c.740G > A, c.852C > G	p.Trp247*, p.Tyr284*	Recessive, compound heterozygous	Bell et al., 2019	5 months	Female	No	Yes	Yes	Yes	No	No	Unknown	No	
c.820C > T	p.Gln274*	Homozygous non-sense	Fichera et al., 2019	4 and 10 years	Male, female	No	No	Yes	Yes		Yes	Unknown	No	
c.892C > T	p.R298*	Homozygous truncating	Wenderski et al., 2020	5 years (Male)	3 females, male	Yes	Yes	Yes	Yes	No	Yes	Yes, due to seizures	No	
c.893G > A	p.Arg298Gln	Homozygous missense	Karaca et al., 2015	Unknown	Female, male	Yes	Yes	Yes	Unknown	Unknown	Unknown	Unknown	No	
c.999T > A	p.Cys333*	Homozygous non-sense	Maddirevula et al., 2019	13 months	Female	Unknown	Unknown	Unknown	Yes	Unknown	Yes	Unknown	Yes	Hydrophobic domain
c.1027G > C	p.Gly343Arg	Dominant, *de novo* missense	Bell et al., 2019	12 years	Female	No	Yes	No	Yes	Yes	No	Unknown	No	
c.1027G > A	p.Gly 343Arg	Dominant, *de novo* missense	Bell et al., 2019	3.5, 5.75, 4.5, 3, 21, and 2.5 years	2 males, 6 females	Yes	Yes	1 of 8	Yes	No	Yes	Unknown	No	
c.1045G > A	p.Gly349 Ser	Homozygous missense	Bell et al., 2019; Fichera et al., 2019	4, 5, and 6 years	3 females	No	Yes	Yes	Yes	No	Yes	Unknown	Yes	Glycine at position 349 is highly conserved
c.1177G > A	p.G393R	Homozygous missense	Wenderski et al., 2020	1 year, 4 years	Male, female	Yes	Yes	Yes	Yes	Yes	Yes	Unknown	No	
c.1231C > T, c.669 + 1G > A	p.Gln411*, splicing	Recessive, compound heterozygous	Bell et al., 2019	12 months	Male	No	Yes	Yes	Yes	Yes	Yes	Yes	No	
c.1249G > T	p.Gly417 Trp	Recessive, homozygous missense	Wenderski et al., 2020	6 years	Male	No	Yes	Yes	Yes	No	Yes	Unknown	No	
c.1261_ 1275del	p.Val421_ Cys425del	Homozygous in-frame deletion	Yüksel et al., 2019	1, 1, and 3 years	4 female, 2 male (3 with confirmed mutation)	No	No	Yes	Yes	Yes	Yes	Unknown	Yes, 3	
c.1279 delT	p.*427 Aspext*32	Homozygous stoploss	Sajan et al., 2017	Unknown	Unknown	Unknown	Unknown	Unknown	Unknown	Unknown	Unknown	Unknown	Unknown	
c.1279 delT	p.*427 Aspext*33	Homozygous stoploss	Bell et al., 2019	4.5 years	Female	No	Yes	Yes	No	Yes	Yes	Unknown	No	

Induced pluripotent stem cell (iPSC) derived neurons from patients with the c.1279del (p.*427Aspext*33) recessive *BAF53B* mutation had almost a complete loss of MAP2 (a marker of neuronal branching) and increased nuclei size at 15DIV (days *in vitro*), similar to that of *BAF53B* KO neurons (Wenderski et al., 2020). Interestingly, this mutation occurs in the final exon of *BAF53B* and protein expression is retained in these cells (Bell et al., 2019). The defects present in neurons with c.1279del phenocopy BAF53B deletion, suggesting they are likely a result of loss of function due to alterations to BAF53B protein structure. However, defects in neuronal dendritic branching are likely transient as at 25DIV, neurons with this mutation appeared similar to wild type, indicating *BAF53B* deficits result in delayed differentiation (Bell et al., 2019). Neurons with the most common dominant *de novo* mutation (1027G > A) did not display defects in *TPPP* (tubulin polymerization-promoting protein) or *FSCN1* (fascin actin-bundling protein 1) expression that were seen in *BAF53B* KO neurons (Bell et al., 2019). This data suggests that dominant and recessive mutations in BAF53B cause disease through unique molecular mechanisms and is consistent with differential symptoms present in patients (Bell et al., 2019).

There are very few reported cases of patients with *BAF53B* mutations, but this number is steadily growing due in large part to increased clinical use of whole exome and whole genome sequencing. The heterogeneity and overlap of symptoms with ID, epilepsy and ASD may have hindered the identification of mutations in *BAF53B*. An example of this is the eventual identification of the *BAF53B* homozygous stoploss mutation c.1279delT (p.*427Aspext*32) in a cohort of Rett Syndrome patients (Sajan et al., 2017). Wenderski et al. (2020) examined whole exome sequencing on 135 ASD patients and identified 13 children from 6 families with homozygous recessive mutations in *BAF53B* (Figure 1 and Table 1). In addition to symptoms of ASD, these children with *BAF53B* mutations demonstrated ID, epilepsy, corpus callosum hypoplasia, developmental delay and hyperactivity (Wenderski et al., 2020). iPSCs derived from patients with mutations located in residues essential for stabilizing the binding surface between BAF53B and BRG1 (c.460C > T;p.Leu154Phe and c.1177G > A;p.Gly393Arg), had severe reductions in BAF53B protein (Wenderski et al., 2020). The mutant protein failed to incorporate into the BAF complex and resulting in defective nBAF complexes lacking BAF53B (Wenderski et al., 2020). This data further supports the idea that recessive mutations that occur before the final exon of the *BAF53B* coding region result in mRNA destabilization and non-sense mediated decay.

## BAF53B Regulates Neuronal Development and Behavior

To study the function of Baf53b, several mouse models have been used. Wu et al. (2007), were the first to generate mice lacking exon 2 and 3 of *Baf53b* by homologous recombination (Baf53b^–/–^ mice). Baf53b^–/–^ mice were born at expected Mendelian ratios but only 25% survived beyond postnatal day 2 and 12% to adulthood (Wu et al., 2007). Despite the brain of Baf53b^–/–^ mice appearing grossly normal, hippocampal Baf53b^–/–^ neuron cultures had severe defects in activity-dependent dendrite growth (Wu et al., 2007). RNAi knockdown of Brg1, Baf57, and Baf45b in Baf53b^–/–^ neurons did not exacerbate dendritic growth defects (Wu et al., 2007), indicating Baf53b is essential for nBAF’s regulation of activity-dependent dendrite growth. The defect in activity-dependent outgrowth is Baf53b specific, as reintroduction of Baf53b not Baf53a was able to rescue dendrite branching (Wu et al., 2007). When investigating the consequences of Baf53b deletion *in vivo*, the apical dendrites of layer 5 pyramidal neurons had a reduction in oblique branches (Wu et al., 2007). Additionally, similar to patients with mutations in *BAF53B*, postnatal mice lacking Baf53b had a 1.7-fold reduction in the percent of myelinated axons in the hippocampal alveus (Wu et al., 2007). As Baf53b is not expressed in oligodendrocytes, the cells that produce myelin, it is likely that defective myelination is due to the interaction between neurons and oligodendrocytes.

Remarkably, backcrossing surviving Baf53b^–/–^ mice to either C57BL/6 or 129S6/SvEv strains promotes survival and Baf53b^–/–^ mice then survive to adulthood when supplemented after weaning (Wenderski et al., 2020). Focusing on ASD-related behaviors, Baf53b^–/–^ mice had decreased social interaction in the juvenile interaction test and three-chamber sociability assay (Wenderski et al., 2020; Walsh et al., 2021). Furthermore, Baf53b^–/–^ mice had significantly increased stereotypic counts as measured by repeated breaks of a photo beam in the open field test (Wenderski et al., 2020). In neuropsychiatric disorders, spatial, working and fear memories are the most commonly affected, making them important to study in animal models (Gallo et al., 2018). Baf53b^–/–^ mice also showed spatial and working memory impairments in the hippocampal-dependent Barnes maze and the prefrontal cortex-dependent T-maze (Wenderski et al., 2020). Patients with *BAF53B* mutations often exhibit hyperactivity (Bell et al., 2019), and this symptom is recapitulated in Baf53b^–/–^ mice as they exhibit three times greater distance traveled in the open field test (Wenderski et al., 2020).

As there are clear differences in the diagnosis of ASD between sexes (Loomes et al., 2017) it is important to analyze behaviors with sex as a biological factor. Female Baf53b^–/–^ mice, but not male Baf53b^–/–^ mice had increased anxiety (Wenderski et al., 2020). Of note, out of those reporting, 66% of patients with *BAF53B* mutations were female (29/44) (Bell et al., 2019; Fichera et al., 2019; Maddirevula et al., 2019; Wenderski et al., 2020). Enhancement of serotonin signaling rescued social defects and hyperactivity, but not memory deficits seen in Baf53b^–/–^ mice (Walsh et al., 2021) suggesting ASD-like features may be independent of memory deficits. Together, these data indicate Baf53b^–/–^ mice model ASD-like behaviors and ID similar to that of patients with BAF53B mutations.

## BAF53B Regulates Adult Long-Term Memory

To circumvent the lethality of complete *Baf53b* deletion and to study the role of Baf53b in the adult brain, Vogel-Ciernia et al. (2013) examined Baf53b heterozygous null mice (*Baf53b*^ +/−^). *Baf53b*^ +/−^ mice have normal motor function and levels of anxiety, making them a good candidate to study the role of Baf53b in transcriptional regulation underlying adult long-term memory formation (Vogel-Ciernia et al., 2013). Consistent with a requirement for new gene expression during learning and Baf53b’s potential role in activity-dependent transcription, *Baf53b*^ +/−^ mice had significant impairments in long-term object location (OLM), object recognition (ORM), and contextual fear memory (Vogel-Ciernia et al., 2013). Reintroduction of Baf53b into the dorsal hippocampus rescued defects in long term OLM (Vogel-Ciernia et al., 2013), supporting the reversibility of the phenotype. Baf53b is also required for cocaine-induced conditioned place preference (CPP) (White et al., 2016), indicating Baf53b plays an important role in the nucleus accumbens. The rescue of social deficits in Baf53b^–/–^ mice with the serotonin receptor 5-HT1b agonist (Walsh et al., 2021) could be due to elevated serotonin levels in the nucleus accumbens as oxytocin-induced release of serotonin is critical for social reward (Dölen et al., 2013).

In addition to examining Baf53b^ +/−^ mice, two different types of dominant negative transgenic mice that over-express a mutant form of Baf53b have been developed. Vogel-Ciernia et al. (2013) over-expressed Baf53b with a deletion in the hydrophobic domain corresponding to amino acids 323–333 (*Baf53bΔHD*). The hydrophobic domain of Baf53b is predicted to form protein-protein interactions with other nBAF subunits and its deletion in Baf53a results in a dominant-negative mutation (Park et al., 2002). There is one human mutation identified thus far within the hydrophobic domain of *BAF53B* (c.999T > A; p.Cys333*) (Maddirevula et al., 2019). The patient (and potentially affected sibling) presented with global developmental delay, agenesis of the corpus callosum, mild atrophic changes and simplified gyral pattern (Maddirevula et al., 2019). In comparison, Ciernia et al. (2017) generated transgenic mice that over-express a mutant form of Baf53b with a deletion of the entire SB2 (BAF53bΔSB2) (Ciernia et al., 2017). There has been one missense mutation uncovered in subdomain 2 of BAF53B (c.230A > G;p.Asp77Gly) (Bell et al., 2019). As this region is the most divergent between BAF53B and BAF53A, mutations in this region are compelling. Symptoms of the patient identified include developmental delay, ID and ASD. To date, there have been no studies performed on patient cells with either the HD or SB2 mutations and so their exact molecular functions are not well understood.

In transgenic mice, the *Baf53bΔHD* and *Baf53bΔSB2* transgenes are over-expressed in postnatal forebrain excitatory neurons by the *Camk2a* promoter. The delayed postnatal induction of the transgenes in both models limits their application for early developmental studies, but does allow for examination of Baf53b in adult plasticity. In both cases, two independent lines were established; one expressing high levels of transgene and one expressing low levels to account for off-target impacts of transgene integration. Similar to *Baf53b*^ +/−^ mice, *Baf53bΔHD* mice, and *Baf53bΔSB2* mice showed defects in long term OLM, ORM, and long-term contextual fear memory, but not short term memory (Vogel-Ciernia et al., 2013). Cocaine-induced CPP was also examined in *Baf53bΔHD* mice to determine if *Baf53b* mutation results in abnormal reward induced memory (White et al., 2016). Indeed, *Baf53bΔHD* results in defective cocaine CPP in a dose-dependent manner (White et al., 2016), suggesting Baf53b plays a critical role in drug-context associations.

Baf53b also contributes to the establishment of long-term fear memory. Endogenous Baf53b is upregulated in the lateral amygdala 2 days after auditory fear conditioning (Yoo et al., 2017, 2020). Acute knock down of *Baf53b*, specifically in the lateral amygdala, produced robust deficits in long-term cued fear conditioning (Yoo et al., 2017). Impairments in fear conditioning were specific to Baf53b knockdown as deletion of the nBAF subunit Baf45b, had no effect on memory (Yoo et al., 2020). In support of this, Baf53b and a chimeric Baf53a containing the SB2 domain of Baf53b were able to enhance fear memory (Yoo et al., 2017). Together data from multiple Baf53b manipulations demonstrates Baf53b is essential for long-term memory and social behaviors that could be relevant to ASD patients.

## BAF53B Regulates Synaptic Plasticity

The severe deficits in dendritic branching observed by Wu et al. (2007) supports the important role of Baf53b in regulating neuronal connectivity and circuit formation. To functionally examine the impacts on neuronal connectivity and plasticity, several studies have examined electrophysiological recordings of neuronal activity in Baf53b mutant mice. Long-term potentiation (LTP) is the electrophysiological correlate of long-term memory and its stable induction and maintenance requires significant remodeling of neuronal synapses (Toni et al., 2001). Maintenance, but not induction, of hippocampal slice LTP was significantly impaired in *Baf53b*^ +/−^ mice and in the presence of either low or high levels of the Baf53bΔHD transgene (Vogel-Ciernia et al., 2013). However, high levels of Baf53bΔHD produced increased initial potentiation suggesting the dosage of transgene expression affects the induction of LTP (Vogel-Ciernia et al., 2013). Defects in CPP and LTP in the nucleus accumbens can be reversed in *Baf53bΔHD* mice when given recombinant brain derived neurotrophic factor (BDNF) (White et al., 2016) which is also decreased following Brg1 knockout in neurons (Zhang et al., 2015).

LTP stabilization requires synaptic remodeling through activation of actin treadmilling, a process regulated by phosphorylation of cofilin at activated dendritic spines (Chen et al., 2007). LTP induction in B*af53b*^ +/−^ (Vogel-Ciernia et al., 2013) or *Baf53bΔSB2* (Ciernia et al., 2017) hippocampal slices failed to fully engage cofilin at PSD95 positive dendritic spines, suggesting a failure in actin remodeling may underlie LTP and memory deficits in Baf53b mutant animals. In support of this hypothesis, introduction of phosphomimic cofilin protein using adeno-associated viral over-expression in brain, rescues LTP and long-term memory deficits in *Baf53bΔSB2* mice (Ciernia et al., 2017). Baf53b does not appear to directly regulate cofilin mRNA expression (Vogel-Ciernia et al., 2013) and how nBAF directly or indirectly controls the actin signaling pathway remains to be uncovered.

Impairments in LTP and dendritic spine plasticity suggest that Baf53b regulated gene expression may alter the basic structure of dendritic spines. When looking at the number and structure of dendritic spines in juvenile *BAF53ΔHD*^high^** mice, there was a modest decrease in the overall spine density with a significant increase in mushroom spines, and a concomitant decrease in thin spines of CA1 pyramidal cells (Vogel-Ciernia et al., 2013). However, the opposite shift in spine ratio was seen in juvenile hippocampus following Brg1 knockdown (Zhang et al., 2015) and adult *Baf53bΔSB2* mice do not show deficits in the number or type of dendritic spines (Ciernia et al., 2017). Additionally, there were no defects in spine density in neurons of the Baf53b^–/–^ lateral amygdala, however, transient expression of wild type Baf53b produced an increase in total spine density, specifically thin-type spines, following fear conditioning (Yoo et al., 2017). The impact on dendritic spines may be dependent on developmental stage at which nBAF is impaired and/or dependent on the type of nBAF manipulation. Bap55, the Drosophila ortholog of Baf53b, has been shown to be crucial for dendritic targeting of neurons in the fly olfactory system (Tea and Luo, 2011). Baf53b was able to rescue deficits in dendrite targeting (Tea and Luo, 2011; Wenderski et al., 2020) but not Baf53b containing patient missense variants c.460C > T (p.Leu154Phe) or c.1177G > A (p.Gly393Arg) (Wenderski et al., 2020). Together, these models provide compelling evidence that Baf53b is critical for neuronal development and synaptic plasticity.

## The Role of BAF53B in Regulating Gene Expression

As a chromatin remodeling complex, it is expected that nBAF controls a transcriptional program required for dendrite outgrowth and synapse formation. Transcriptional levels of many genes involved in neurite outgrowth including *Gap43*, *Stmn2*, *Rap1A*, and *Gprin1*, were altered in Baf53b^–/–^ neurons (Wu et al., 2007). Additionally, several Rho-GTPases important for activity-dependent dendrite development, were aberrantly expressed in Baf53b^–/–^ neurons (Wu et al., 2007). Reintroduction of the depleted Rho-GEF Ephexin1, rescued the impaired activity-dependent dendritic outgrowth in Baf53b^–/–^ neurons, suggesting it is an essential nBAF target gene (Wu et al., 2007). A chimeric Baf53a containing the SB2 domain of Baf53b was able to rescue dendritic branching and *Ephexin1* and *Gap43* expression in Baf53b^–/–^ neurons (Wu et al., 2007), highlighting the importance of SB2 of Baf53b.

To investigate potential nBAF-regulated target genes involved in memory and synapse formation *in vivo*, RNA-sequencing (RNA-seq) was performed on the dorsal hippocampus following a learning event in *Baf53b*^ +/−^ adult mice (Vogel-Ciernia et al., 2013). There were several genes that failed to increase following learning in *Baf53b*^ +/−^ mice that were involved in chromosome organization and chromatin modification, pointing to a role for Baf53b in higher-order chromatin structure (Vogel-Ciernia et al., 2013). Additionally, genes involved in postsynaptic cell membrane and cytoskeleton regulation failed to show an activity-dependent decrease in *Baf53b*^ +/−^ brain tissue (Vogel-Ciernia et al., 2013). Focusing on genes involved in spine plasticity, several genes involved in phosphorylation of cofilin and cytoskeleton reorganization were misregulated in *Baf53b*^ +/−^ brain (Vogel-Ciernia et al., 2013). Additionally, following deletion of Brg1 in neurons of the dentate gyrus, several genes involved in cytoskeleton, synaptogenesis, and calcium signaling were misregulated (Zhang et al., 2015). Comparing Brg1-regulated genes to known synaptic genes and genes linked to ASD, there was significant overlap, providing a potential link between defective synaptic plasticity and ASD pathogenesis (Zhang et al., 2015).

Baf53b is required for activity-dependent dendrite branching, memory formation and LTP (Vogel-Ciernia and Wood, 2014) all of which requires *de novo* transcription (Alberini and Kandel, 2015). Baf53b may be involved in the transcription of IEGs in response to neuronal activation, however, the current data is somewhat conflicting. A time course of KCl treatment administered to Brg1 depleted neurons revealed a lack of induction of the IEGs *c-fos* and *Arc* most notably 2 h post activation (Kim et al., 2021). In contrast, in Baf53b^–\–^ neurons where neuronal firing was artificially silenced by tetrodotoxin, IEG expression was aberrantly induced (Wenderski et al., 2020). In fact over 40% of differentially expressed genes were activity response genes that normally would be induced upon neuronal depolarization in wild type cultures (Wenderski et al., 2020). Surprisingly, Baf53b^–\–^ neurons artificially stimulated with KCl also had hyper-induced expression of activity-regulated genes (Wenderski et al., 2020), indicating Baf53b deletion increases IEG expression irrespective of stimulation. Lastly, following a learning event *in vivo*, Baf53b^ +/−^ mice do not show changes in the expression of most IEGs (Vogel-Ciernia et al., 2013). This discrepancy could be due to the heterogeneous composition of brain tissue, the artificial nature of culture conditions required for silencing or depolarizing entire neuronal populations, or differences in heterozygous vs. homozygous loss of Baf53b. As there have been reports of normal IEG expression (Vogel-Ciernia et al., 2013) and activation (Wenderski et al., 2020) following Baf53b deletion, future work will be required to more fully investigate Baf53b’s role in IEG expression.

Evidence does support the Baf53b-regulation of a subset of activity-dependent genes. RNA-seq in the lateral amygdala following auditory fear conditioning training revealed *Fgf1* as a candidate gene that failed to increase following Baf53b deletion (Yoo et al., 2020). *Fgf1* is normally increased upon neuronal activity (Ma et al., 2009) and is important for neurite outgrowth (Raju et al., 2014) and memory (Uchida et al., 2017). Restoration of FGF1 levels in Baf53b^–/–^ lateral amygdala rescued memory deficits (Yoo et al., 2020). Together, this provides evidence that nBAF is critical for gene expression involved in dendrite outgrowth, synapse formation and maturation, and ultimately memory formation. However, the exact target genes and direct mechanistic linkage to synapse function is still an active area of investigation.

## Potential nBAF Mechanisms for Neuronal Gene Regulation

The BAF complex has been shown to be essential for regulation of gene expression across numerous cell types and contexts (Alfert et al., 2019). *In vitro*, BAF complexes can slide, evict, and unwind nucleosomes as well as participate in DNA looping (Kassabov et al., 2003; Dechassa et al., 2010). Disruption of the BAF complex in cultured cells results in altered nucleosome patterning and loss of nucleosomes at promoters (Tolstorukov et al., 2013). Additionally, BAF has been shown to remodel nucleosomes at enhancers (Hu et al., 2011) and is required at enhancers for genes involved in lineage specification (Alver et al., 2017). The BAF complex directly opposes the PRC deposits the facultative heterochromatin mark H3K27me3 at inactive regions of the genome (Kadoch et al., 2016). While the PRC mediated H3K27me3 deposition is opposed by npBAF to upregulate cell cycle genes (Braun et al., 2021), it is unknown if nBAF acts similarly to increase genes involved in neuronal differentiation. The eviction of the PRC occurs within minutes of BAF complex recruitment and is dependent on the ATPase activity of the BAF complex (Kadoch et al., 2016). In line with this, changes in chromatin accessibility following BRG1/BRM catalytic inhibition occur within 5–10 min and are rapidly restored following inhibitor washout (Schick et al., 2021). Both promoters and enhancers lost accessibility following inhibition of BAF complex ATPase activity (Iurlaro et al., 2021; Schick et al., 2021). These data suggest that the BAF complex is required for establishment and maintenance of accessible chromatin that allows for cell-type specific transcription factor binding (Iurlaro et al., 2021; Schick et al., 2021). This dynamic and activity sensitive system may allow cells to respond to intrinsic and extrinsic signals important for establishing new gene expression depending on the cellular context.

It might be expected that nBAF binding increases global chromatin accessibility, leading to increased expression of genes important for branching and neural activity. However, Brg1 depletion in depolarized neurons did not produce significant changes in chromatin accessibility (Kim et al., 2021). Analysis of chromatin accessibility in silenced Baf53b^–\–^ neurons showed increased accessibility at AP-1 transcription factor motifs. AP-1 is a known regulator of IEG induction in neurons, suggesting that chromatin accessibility at AP-1 regulatory sites is still intact in the absence of Baf53b and may even be enhanced in compensation (Wenderski et al., 2020). Furthermore, BAF mediated changes in chromatin accessibility are not simplistically linked to increases in gene expression, as there are numerous genes that are still increased in expression following depolarization in Brg1 knockout neurons (Kim et al., 2021). Similarly, examination of targeted chromatin looping in prefrontal cortex of Baf53b^ +/−^ mice did not reveal changes in loop formation (Bharadwaj et al., 2014). Overall, the mechanistic link between chromatin structure and nBAF regulation remains largely unclear.

There are currently several potential mechanisms by which nBAF specifically targets neuronal genes. Baf53b may be responsible for recruiting nBAF to target genes, as Baf53b deletion disrupts target promoter binding (Wu et al., 2007). However, Baf53b does not contain a known DNA interacting domain and so presumably any targeting would be via specific interactions with additional transcription factors. For example, BAF complexes are recruited to target genes through interaction with transcription factors like SP1 (Qiu and Ghosh, 2008), MEF2 (Zhang et al., 2015), and AP-1 (Vierbuchen et al., 2017). Myocyte enhancer factor 2 (MEF2) transcription factors regulate several IEGs as well as genes that are mutated in ASD and epilepsy (Flavell et al., 2008). MEF2C is important for dendritic spine and synapse elimination thereby facilitating learning and memory by negative regulation of synapse number (Barbosa et al., 2008). Brg1 regulates several MEF2 target genes including those necessary for synapse structure and plasticity like *Bdnf* and *Nr4a1* (Zhang et al., 2015). Furthermore, MEF2C interacts with Brg1 and is required for its recruitment to activity regulated genes including *Bdnf* and *Arc* (Zhang et al., 2015) making MEF2C a promising nBAF interacting transcription factor.

nBAF may also be targeted by interactions with modified histones. Brg1 contains a bromodomain that binds H3K27ac (Filippakopoulos et al., 2012) and inhibition of this domain results in lower Brg1 binding and target activity-regulated gene expression (Kim et al., 2021). Inhibition of the bromodomain alone did not reduce H3K27ac shortly after depolarization, but inhibition of the histone acetyltransferases p300/CBP did (Kim et al., 2021). As p300/CBP inhibition also blocked Brg1 binding, this suggests H3K27ac recruits BAF to activity acetylated genes (Kim et al., 2021). Several activity-regulated genes showed co-occupancy of Brg1 and H3K27ac at enhancers, including *c-fos* and *Nr4a1* (Kim et al., 2021). Furthermore, enhancer RNAs for *c-fos* and *Arc* activation are decreased following Brg1 knockdown, as was enhancer-promoter looping (Kim et al., 2021). Together, this suggests that gene specific targeting of Baf53b and the nBAF complex may involve several mechanisms working in concert.

nBAF regulation also appears to occur at the level of post-translational modification of its subunits. Brg1 undergoes activity induced phosphorylation at S1382 both *in vitro* and *in vivo* (Kim et al., 2021), which may facilitate an activity-dependent role for the BAF complex. S1382 is located in the histone-interacting Snf2 ATP Coupling (SnAC) domain which regulates ATPase activity and serves as a histone anchor (Sen et al., 2013). Phosphorylation at Brg1 S1382 does not affect its ATPase activity or complex assembly (Kim et al., 2021). However, constitutive phosphorylation of Brg1 S1382 rescued *c-fos* and *Arc* expression in depolarized Brg1^–/–^ neurons (Kim et al., 2021), indicating phosphorylation is required for their expression. Brg1 phosphorylation appears to be necessary for induction of several neuronal IEGs. RNA-seq in neurons containing a non-phosphorylatable Brg1 S1382 showed altered expression of IEGs, which could be rescued with constitutive phosphorylation at S1382 (Kim et al., 2021). In line with this, H3K27ac and enhancer-promoter looping at *c-fos* were enhanced with constitutive Brg1 S1382 phosphorylation (Kim et al., 2021). Brg1 phosphorylation at S1382 and enhancer regulation can modify nBAF’s response to neuronal activity, supporting an additional activity-dependent level of nBAF regulation.

A dual role for the nBAF complex in both transcriptional activation an repression has recently been proposed (Wenderski et al., 2020; Kim et al., 2021). The duality may by conferred in part by the complex’s interaction with either histone deacetylases (HDACs) or histone acetylate transferases (HATs). HDACs remove histone acetyl groups and generally facilitate repression of gene expression while HATs add acetyl groups to histone tails, facilitating gene expression (Vogel-Ciernia and Wood, 2012). Brg1 binds three histone deacetylases Hdac1, Hdac2, and Hdac3 (Qiu and Ghosh, 2008). Proteomic analysis revealed four subunits of the NuRD co-repressor complex (Gata2b, Mta1, Mta3, and Hdac2) were bound to the BAF complex and released upon neuronal stimulation (Kim et al., 2021). In the absence of Brg1 S1382 phosphorylation, more NuRD subunits were co-immunoprecipitated with Brg1 and the KCl-induced departure of the NuRD complex at *c-fos* and *Arc* promoters was impaired (Kim et al., 2021). This data provides compelling evidence that phosphorylation of Brg1 at S1382 provides a precise control mechanism to maintain a repressed state at baseline and to induce the rapid expression of activity-regulated genes following phosphorylation. Additionally, in conjunction with Hdac1, Brg1 was found to bind and repress the *c-fos* promoter under basal conditions (Qiu and Ghosh, 2008). After neuronal depolarization with KCl, Hdac1 is released from the BAF complex and replaced by the HAT CBP to activate *c-fos* expression (Qiu and Ghosh, 2008). These two opposing BAF interacting partners could also explain the discrepancy in activation/inhibition of IEGs in nBAF knockout models. Hdac3 depletion has been shown to enhance memory formation by allowing sustained activation of the IEG *Nr4a2* (McQuown et al., 2011). *Nr4a2* is reduced in KCl stimulated neuronal cultures lacking Brg1 (Zhang et al., 2015). Additional deletion of Hdac3 in the dorsal hippocampus of *Baf53bΔSB2* mice restores memory and LTP impairments (Shu et al., 2018) potentially by elevating *Nr4a2* expression. Together, these findings support the role for additional epigenetic mechanisms such as histone acetylation in dictating nBAF’s regulation of gene expression.

## Conclusion and Future Directions

**FIGURE 2:**
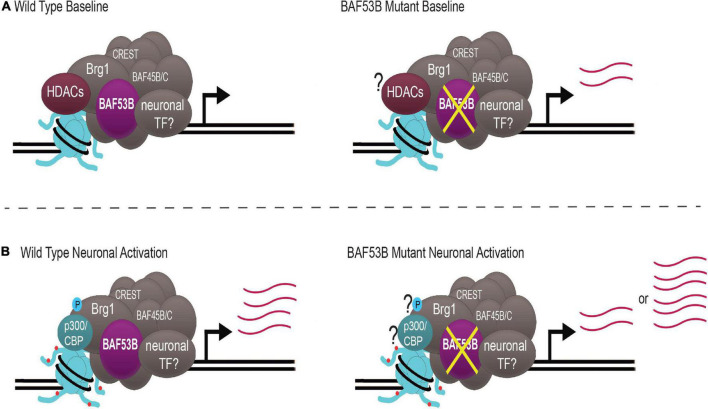
nBAF regulates activity-induced gene expression through a dynamic system. **(A)** Under wild type baseline conditions, HDACs (maroon) are bound to nBAF (gray), which suppresses transcription of target genes. When BAF53B (purple) is mutated, there is atypical upregulation of activity-dependent genes. **(B)** With neuronal activation, Brg1 S1382 becomes phosphorylated (blue circle), p300/CBP (teal) replaces HDACs and H3K27ac (red circles) is increased, facilitating upregulation of activity-dependent genes. When BAF53B is mutated, there are some reports of normal activation of activity-regulated genes and some reports of hyper-induction of activity-regulated genes. Question marks represent key contributors whose status is unknown in BAF53B mutants. Transcript abundance is depicted by pink lines. nBAF specific subunits BAF53B, BAF45B/C and CREST are highlighted.

Recently, whole exome sequencing has identified mutations in *BAF53B* present in patients with DEE76 and ASD. Until these reports, *BAF53B* had not been conclusively reported to play a role in human disease. Baf53b mutant mice display hyperactivity and impairments in long-term memory and social interactions, mimicking behaviors seen in patients. Baf53b regulates activity-dependent dendritic outgrowth and synaptic plasticity, potentially explaining behavioral impairments. As a chromatin remodeling complex, nBAF likely modulates several genes crucial for these processes. nBAF may be important for the expression of activity-induced IEGs, although the data is far from conclusive. It appears as though nBAF is essential for the activation of a subset of IEGs important for memory, including *c-fos*. Inconsistency among reports supports the idea that nBAF may regulate gene expression through a dynamic system that is essential for modulating gene expression quickly and depending on the cellular environment. This is achieved by post-translational phosphorylation of Brg1, as well as nBAF complex interaction with HDACs and HATs (Figure 2).

It is clear that more work is needed to parse out the details of this system and how it might relate to patients with Baf53b-related neurodevelopmental disorders. Nonetheless, Baf53b presents as promising candidate gene as it intersects the two most commonly disrupted systems in ASD, synapse regulation and chromatin remodeling. Recent therapeutic work has focused on developing gene therapies for genetic forms of neurodevelopmental disorders including advances for Rett syndrome (Sinnett et al., 2021), Angelman syndrome (Wolter et al., 2020), and fragile X syndrome (Lee et al., 2018). Baf53b and other nBAF subunits may be amenable to similar types of approaches to directly rescue nBAF function and developmental gene expression. Given that re-expression of Baf53b in heterozygous knockout mice was able to rescue synaptic plasticity and memory deficits (Vogel-Ciernia et al., 2013), gives hope that at least some of the impacts of Baf53b loss during development may be reversible later in life. Alternatively, manipulations of cytoskeleton remodeling machinery (Ciernia et al., 2017) or expression of *Bdnf* (White et al., 2016) can rescue memory deficits and treatment with serotonin rescues social behavior deficits in Baf53b mutant mice (Walsh et al., 2021). Together, this work provides a number of novel avenues to pursues for therapeutic advances for treating ASD symptoms in children with Baf53b and nBAF mutations.

## Author Contributions

MR and AC conceived of the main topic ideas and edited the manuscript. MR wrote the majority of the sections with direct assistance from JJ and TO. All authors contributed to the article and approved the submitted version.

## Conflict of Interest

The authors declare that the research was conducted in the absence of any commercial or financial relationships that could be construed as a potential conflict of interest.

## Publisher’s Note

All claims expressed in this article are solely those of the authors and do not necessarily represent those of their affiliated organizations, or those of the publisher, the editors and the reviewers. Any product that may be evaluated in this article, or claim that may be made by its manufacturer, is not guaranteed or endorsed by the publisher.
